# Induction of Golli-MBP Expression in CNS Macrophages During Acute LPS-Induced CNS Inflammation and Experimental Autoimmune Encephalomyelitis (EAE)

**DOI:** 10.1100/tsw.2007.251

**Published:** 2007-11-02

**Authors:** Tracey L. Papenfuss, J. Cameron Thrash, Patricia E. Danielson, Pamela E. Foye, Brian S. Hllbrush, J. Gregor Sutcliffe, Caroline C. Whitacre, Monica J. Carson

**Affiliations:** ^1^ The Ohio State University, USA; ^2^ University of California Berkeley, USA; ^3^ Scripps Research Institute, USA; ^4^ ModGene, LLC, USA; ^5^ University of California Riverside, USA

**Keywords:** microglia, macrophages, neuroinflammation, autoimmunity

## Abstract

Microglia are the tissue macrophages of the CNS. Microglial activation coupled with macrophage infiltration is a common feature of many classic neurodegenerative disorders. The absence of cell-type specific markers has confounded and complicated the analysis of cell-type specific contributions toward the onset, progression, and remission of neurodegeneration. Molecular screens comparing gene expression in cultured microglia and macrophages identified Golli-myelin basic protein (MBP) as a candidate molecule enriched in peripheral macrophages. *In situ* hybridization analysis of LPS/IFNg and experimental autoimmune encephalomyelitis (EAE)–induced CNS inflammation revealed that only a subset of CNS macrophages express Golli-MBP. Interestingly, the location and morphology of Golli-MBP+ CNS macrophages differs between these two models of CNS inflammation. These data demonstrate the difficulties of extending *in vitro* observations to *in vivo* biology and concretely illustrate the complex heterogeneity of macrophage activation states present in region- and stage-specific phases of CNS inflammation. Taken altogether, these are consistent with the emerging picture that the phenotype of CNS macrophages is actively defined by their molecular interactions with the CNS microenvironment.

## INTRODUCTION

The mammalian CNS is populated very early in prenatal development by tissue macrophages[1,2,3,4]. By adulthood, this CNS-resident population of macrophages, referred to as microglia, are found in all regions of the brain and spinal cord, and comprise ~10–15% of the total cells found in the CNS[1,2,3,4]. Despite a century of study, the *in vivo* function of microglia is still a subject of debate. *In vitro* studies have conclusively demonstrated the neurodestructive potential of microglia when activated by pathogenic stimuli[1,2,3,4]. Conversely, the conservation of the relative concentration and distribution of microglia throughout the CNS across all mammalian species so far examined suggests an important evolutionarily conserved function for these cells in maintaining CNS function. As yet, a viable (spontaneous or transgenically engineered) mammalian model that lacks all microglia does not exist. The absence of such a microglial knockout model has prevented conclusive and direct identification of the necessary and nonredundant neurosupportive functions provided by CNS-resident microglia.

An additional complication confounding this debate is that the CNS is served by at least two types of macrophage populations. One population is defined by a $CD45^{lo}$ phenotype, is located predominantly in the CNS parenchyma, and appears to be rarely replenished from hematogenously derived cells in the mature adult[1,2,3,4]. The second population of cells is defined by a $CD45^{hi}$ phenotype; is located predominantly in perivascular regions, meninges, and choroids plexus; and is frequently replaced by hematogenously derived cells every few weeks[1,2,3,4]. The latter population is alternatively referred to as perivascular microglia or perivascular macrophages, while the former is most commonly referred to as parenchymal microglia. The absence of cell-type specific markers able to distinguish these two populations in histological analysis of CNS pathologies has hindered the definition of their relative roles. Consequently, many investigators choose to refer to all macrophages located within the CNS as microglia, even including those infiltrating from the bloodstream[5].

Three types of studies suggest that both types of microglia (long-term residents and acutely infiltrating cells) can play neuroprotective functions and that the source of microglia may determine their functions. For example, op/op mice are deficient in macrophage-colony stimulating factor (M-CSF) and consequently have ~30–40% fewer macrophages in all organs, including the CNS[6,7]. While the CNS of op/op mice appears histologically indistinguishable from wild-type mice, op/op mice display severe neurological deficits, including impaired auditory and visual processing, with specific abnormalities associated with cerebral cortex function[7]. In addition, M-CSF enhances neuronal process extension *in vitro*, but only in cultures with M-CSF receptor–expressing microglia[6]. Interestingly, while wild-type mice do not develop spontaneous, nontransgenically induced features of Alzheimer's disease, op/op mice have been reported to develop A-beta plaques spontaneously[8]. Injection of M-CSF not only increases the numbers of CNS microglia by nearly 70%, but it also decreases the number of amyloid plaques found in the op/op CNS. While these data suggest that CNS macrophages are essential for optimal neuronal development and function, they do not conclusively reveal the relative importance or roles of the two types of CNS macrophages.

The human syndrome called Nasu-Hakola's disease provides another compelling example that CNS macrophages contribute to optimal neurological function[9,10]. Individuals with Nasu-Hakola's disease lack a functional TREM2 pathway and develop early-onset cognitive dementia in their 20s and 30s. Surprisingly, within the CNS, only microglia express detectable TREM2 mRNA[11,12]. In addition, CNS-resident ($CD45^{lo}$) microglia express ~10-fold higher levels of TREM2 than acutely infiltrating ($CD45^{hi}$) macrophages[11,13]. These data suggest that aberrant microglial function can trigger clinical symptoms normally associated with a primary dysfunction in CNS neurons!

Finally, *in vivo* studies using irradiation bone marrow chimeras suggest that both acutely infiltrating macrophages and long-term CNS-resident cells each can play distinct and nonredundant neuroprotective functions. In irradiation bone marrow chimeric mice, the host bone marrow is killed by irradiation and replaced by genetically distinct donor bone marrow[4]. Using this method, Rivest and colleagues demonstrated that hematogenously derived macrophages were more phagocytic than CNS-resident microglia[5]. Additionally, they found that the failure to retain or to recruit continually these donor-derived macrophages to the CNS correlated with increased amyloid plaque deposition in murine models of Alzheimer's disease[5]. Using similar methods, Byram et al. demonstrated that peripheral myeloid cells were essential to initiate protective T-cell responses inhibiting motoneuron death following facial axotomy[14]. However, Byram et al. also demonstrated that CNS-resident cells were absolutely essential to evoke or sustain the neuroprotective response; acutely infiltrating macrophages alone were insufficient.

Taken together, these data illustrate that all populations of CNS macrophages are likely to play specific and essential functions to maintain optimal neuronal function. The implication of these data is that dysfunction in any CNS macrophage compartment also has the potential to facilitate or directly contribute to the onset and progression of clinical CNS neurodegeneration. Effective and targeted therapeutic intervention for CNS neurodegenerative diseases is thus likely to depend on identifying the relative contributions of each CNS macrophage population as a function of defined stages of each type of CNS neurodegeneration. As a first step toward this goal, we and others have conducted gene-profiling screens to define molecules that are enriched in each cell type[11,13,15,16]. Here we characterize the induced expression of a specific Golli-myelin basic protein (Golli-MBP), referred to as BG21, which was identified by our screens as being regulated by inflammatory signals and enriched in peripheral macrophage populations[17].

The classic MBPs are produced primarily by CNS oligodendrocytes[17,18]. Within the CNS, the classic forms of MBPs function to maintain CNS myelin structure. Low, but detectable, expression of classic MPBs can also be detected in several sites outside the CNS, including peripheral myelin and the thymus[17,18]. Golli-MBPs are not structural myelin proteins[17], however, they do share exons and antigenic protein domains with classic MBPs due to alternative splicing of MBP exons into a transcript initiated by the Golli-promoter upstream from the classic MBP promoter (Fig. 1a)[17]. Golli-MBPs are expressed by neurons, oligodendrocytes, as well as a wide variety of immune cells, including most peripheral macrophage populations[19,20]. High doses of LPS also have been reported to induce Golli-MBP expression in rodent neonatal and human fetal cultured microglia[21]. In addition, Golli-MBP immunoreactivity has been reported in microglia and/or CNS-infiltrating macrophages surrounding inflammatory lesion in CNS tissue taken from human multiple sclerosis (MS) patients[21].

MS is an autoimmune disorder characterized by targeted inflammation of CNS white matter coupled with oligodendrocyte death and demyelination[22]. Myeloid expression of Golli-MBPs has alternatively been hypothesized to promote destructive inflammatory demyelinating episodes of MS and to promote remission and repair phases of MS[19]. By presenting their own self-expressed MBP peptides to autoreactive MBP-specific T cells, Golli-MBP+ myeloid cells could serve to drive activation of destructive myelin-specific T cells. Conversely, production and secretion of Golli-MBP proteins from cultured microglia does promote the proliferation of oligodendrocyte precursors *in vitro* and in organotypic slice cultures[21]. Thus Golli-MBP production may also serve to remediate the consequences of autoimmune T-cell activation by promoting CNS repair.

As yet, a comparison of Golli-MBP mRNA expression in the healthy vs. inflamed CNS has not been reported. Here we explore the general utility of using Golli-MBP to label CNS macrophage populations in two acute/remitting murine models of CNS inflammation: LPS/IFNg-induced inflammation and experimental autoimmune encephalomyelitis (EAE).

## MATERIALS AND METHODS

### Cells and Tissues

Microglia were isolated by flow cytometry from C57BL/6J neonatal mixed glial culture as previously described using phycoerythrin (PE)-conjugated antibodies directed against Fc receptor (FcR)/CD16/CD32 (Pharmingen, San Diego, CA)[11]. Peritoneal macrophages were isolated from C57Bl/6J mice following thioglycolate injections as previously described[11]. Cells were treated for 1 or 22 h with LPS (100 ng/ml) and IFNg (10 U/ml).

### Northern Blot Analysis poly(A) RNA, 2 µg per lane, was resolved by electrophoresis in a 1.5% agarose/1.2 *M* formaldehyde gel, transferred to nylon membrane, and hybridized with $^{32}$P-radiolabeled probe corresponding to Golli-MBP exon 5a (accession #L07508 nucleotides 414-627) as described previously[11]. Expression of each transcript was quantified using NIH MAGE 1.62 software (National Institutes of Mental Health, Bethesda, MD). The actin loading control for the northern blot depicted in Fig. 1B has been previously published[11].

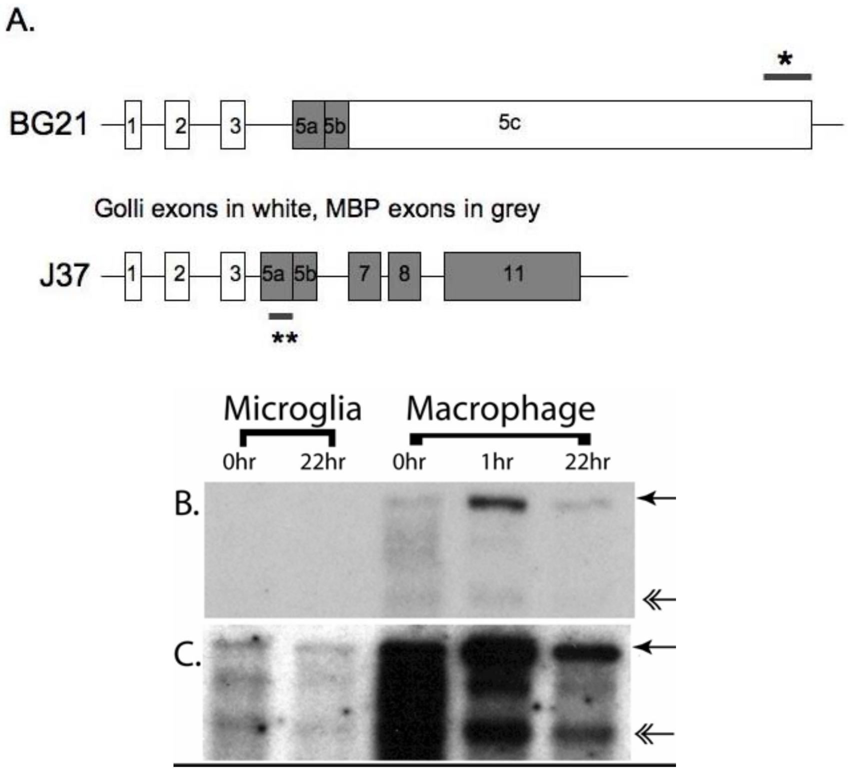

**FIGURE 1**. Golli-MBP expression is enriched in culture macrophages as compared to cultured microglia. (A) The exon structures of two Golli-MBP transcripts are schematically represented. Exons encoding Golli-specific amino acids are depicted in white, exons encoding “classic” MBP amino acids are depicted in grey. The single asterisk/grey line identifies the sequence from exon 5c used for *in situ* hybridization probes (Figs. 2 and 3). The double asterisk/grey line identifies the sequence from exon 5a used for northern blot probes (Fig. 1B, C). (B and C) Northern blot analysis of mRNA isolated from microglia, purified by flow cytometry from mixed glial cultures and thioglycolate-elicited peritoneal macrophages. Cells were either untreated or treated for 22 h with LPS/IFNg. The dark single arrow indicates the 5.1-Kb BG21 transcript, the double arrow indicates the 2.6-Kb J37 transcript. “B” represents a 16-h exposure and “C” represents a 2-week exposure of the same northern blot.

## Mice

LPS (100 ng/ml) and IFNg (10 U/ml) were injected intracerebrally into the striatum of C57Bl/6J mice in a total volume of 5 µl after mice had been anesthetized with metofane. Mice were euthanized at 0, 6hrs, 1 day and 3 days after LPS/IFNg injection, and their CNS was fixed with 4% paraformaldehye by intracardiac perfusion followed by 24-h postfixation.

A MBP-induced form of remitting EAE was induced as previously described[23]. Briefly, B10.PL mice, 6–8 weeks old, obtained from The Jackson Laboratory (Bar Harbor, ME) were injected s.c. over four sites on the flank with 200 µg of MBP in CFA containing 200 µg killed *Mycobacterium tuberculosis*, Jamaica strain. Pertussis toxin (List Biological Laboratories, Campbell, CA), 150 ng in 0.2 ml of PBS, was given i.p. at the time of immunization and 48 h later. Mice with full paralysis of both hind limbs were assigned an acute EAE score of 4 and euthanized. Acute EAE occurred within 14 days of immunization and remission of clinical symptoms occurred within 28 days of immunization. CNS tissue was harvested as described above from three to six mice for each condition and time point.

### *In situ* Hybridization Analysis

*In situ* hybridization was performed on free-floating brain cryosections and on slides mounted for lymph node sections as described previously[11]. Briefly, 25-µm sections were hybridized overnight at 55°C with $^{33}P$-labelled riboprobes ($10^7$ cpm/ml) corresponding to the final 400 nucleotides of the untranslated region in Golli-MBP exon 5c. Myeloid cells and blood vessels were visualized with biotinylated tomato lectin (Sigma) and streptavidin-horseradish peroxidase. After dipping in Ilford K-5 emulsion (Polysciences, Warrington, PA), slides were exposed for 4 weeks and developed with Kodak D19 developer (Fisher Scientific). Nuclei were counterstained with Mayer’s hematoxylin.

## RESULTS AND DISCUSSION

### Golli-MBP Expression is Expressed at Much Higher Levels in Macrophage than Microglia *In Vitro*

In the mouse, two Golli-MBP transcripts have been reported that contain both Golli-specific and MBP domains: BG21 and J37 (Fig. 1A)[18]. The BG21 transcript encodes Golli amino acids 1-133 spliced to MBP amino acids 1-56 spliced to five additional Golli-specific amino acids. J37 transcript encodes Golli amino acids 1-133 spliced to MBP amino acids 1-102 spliced to MBP amino acids 155-168. On the RNA level, exon 5A is present in both transcripts, while exon 5c is present in only BG21. As part of our ongoing studies to define cell-type specific molecular fingerprints, we compared the expression of BG21 and J37 in primary microglia and thioglycolate-elicted peritoneal macrophages.

In Fig. 1, the same northern blot has been exposed for a short (Fig. 1B,16 h) and long (Fig. 1C, 2 week) time periods so that the expression of both the 5.1-Kb BG21 and the 2.6-Kb J37 transcripts could be compared between all of the microglia and macrophage samples. In general, Golli-MBP expression was ~10-fold higher in all macrophages (treated and untreated) than in microglia. In addition, the ratio of BG21:J37 transcripts differed between untreated and LPS/IFNg-treated cells. In untreated microglia and macrophages, the ratio of these two transcripts was ~equivalent; while BG21 levels were 1.5-fold higher than J37 levels in LPS/IFNg-treated microglia and more than 5-fold higher in LPS/IFNg-treated macrophages. In contrast to previous reports, we did not see a sustained increase in Golli-MBP expression, although a transient >5-fold increase in BG21 expression was detected within 1 h of LPS/IFNg treatment.

The difference between our study and previous reports may be due the differences in the doses of LPS used and our detection methods[21]. In our studies, we used 100 ng/ml, which was a 25- to 50-fold lower dose than was required to observe an effect in previous reports. In addition, we examined mRNA production rather than Golli-MBP immunoreactivity.

### Detectable Expression of Golli-MBP is Rare in the CNS of Untreated and LPS-IFNg-Injected Mice

Because BG21 was expressed at a much higher level than J37 in activated macrophages, we chose to focus primarily on characterizing the expression of BG21 by *in situ* hybridization analysis in two different models of CNS inflammation: intracerebral injection of LPS/IFNg and EAE. For these studies, microglia, macrophages, and blood vessels were identified by their ability to bind tomato lectin.

We have previously demonstrated that intracerebral injections of LPS/IFNg cause a robust, but transient, infiltration of activated $CD45^{h}$i macrophages into the CNS and a transient activation of resident $CD45^{lo}$ microglia[11]. Unactivated macrophages *in vitro* expressed high levels of Golli-MBP (Fig. 1B, C), therefore, we examined the brain regions known to be populated by $CD45^{hi}$, hematogenously derived cells; specifically the perivascular regions, the chorioid plexus, and the meninges. We could detect Golli-

MBP expression in many lectin-negative cells, consistent with many previous characterizations of Golli-MBP expression in the CNS[17]. However, we could not detect Golli-MBP mRNA expression in any lectin-positive cells in any brain regions by *in situ* hybridization analysis in the CNS of untreated control mice (data not shown). This is unlikely to be a strain-specific effect as identical results were observed in untreated control tissue C57Bl/6J, BALB/c, B10.D2, and B10.PL mice.

In contrast, Golli-MBP mRNA was clearly detected in small numbers of lectin+ cells by 1 day post-LPS/IFNg injection. These Golli-MBP macrophages were almost always found in close proximity to the injection site, but did not necessarily display the most activated macrophage morphology visible within the same regions (Fig. 2). The limited Golli-MBP expression observed at 1 day post-LPS/IFNg injection is unlikely to reflect limited macrophage infiltration or limited microglial activation. In this same model, we have previously reported that macrophages throughout the entire CNS of injected are rapidly induced to express high levels of interferon response gene 2 (IRG2) by 1 day postinjection[11]. By 3 days postinjection, Golli-MBP expression could no longer be detected in any population of CNS macrophages. This observed loss of Golli-MBP expression in lectin+ cells also correlated with the outflux of acutely infiltrating $CD45^{hi}$ macrophages from the CNS. It is also important to note that we did not observe any change in the level or percentage of Golli-MBP expression in lectin-negative cells as a function of this treatment.

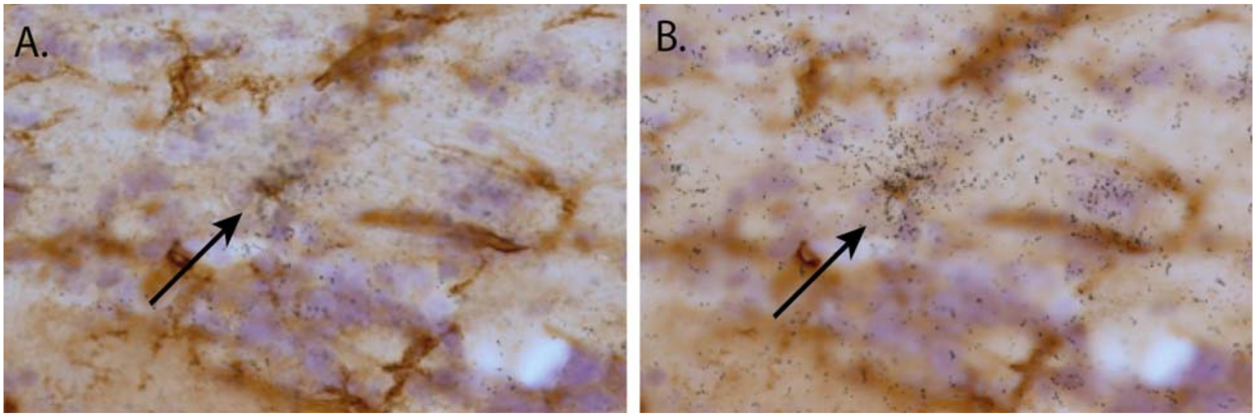

**FIGURE 2**. Golli-MBP expression is detected in only a few CNS macrophages following intracerebral LPS/IFNg injection. Panels A and B represent two different focal planes of the same histological section of the adult murine striatum 24 h following intracerebral injection of LPS/IFNg. Microglia, macrophages, and blood vessels are visualized in brown with tomato lectin, nuclei are visualized in blue with hematoxylin, and BG21 mRNA expression was visualized by photographic emulsion grains (black) with $^{33}P$ label riboprobes. The arrow indicates a Golli-MBP–expressing, lectin+ cell.

## Golli-MBP+ Macrophages are Detected Primarily in Perivascular Sites within the CNS During Acute EAE

Expression of Golli-MBP by peripheral myeloid cells or even CNS-resident microglia has been hypothesized to contribute to susceptibility or exacerbation of CNS autoimmune responses directed against CNS myelin. Here, we examined whether the expression of Golli-MBP mRNA by CNS macrophages correlated either with clinical stage or histopathology in MBP-induced EAE. By *in situ* hybridization analysis, Golli-MBP expression could not be detected in any population of CNS macrophages in the CNS of either untreated mice or MBP-immunized mice while at preclinical stages of EAE (data not shown) consistent with our previous set of experiments. In stark contrast, Golli-MBP mRNA was readily detected in many CNS macrophages scattered throughout the entire cerebellum and spinal cord in mice exhibiting symptoms of acute EAE (characterized by complete paralysis of both hind limbs) (Fig. 3A, B).

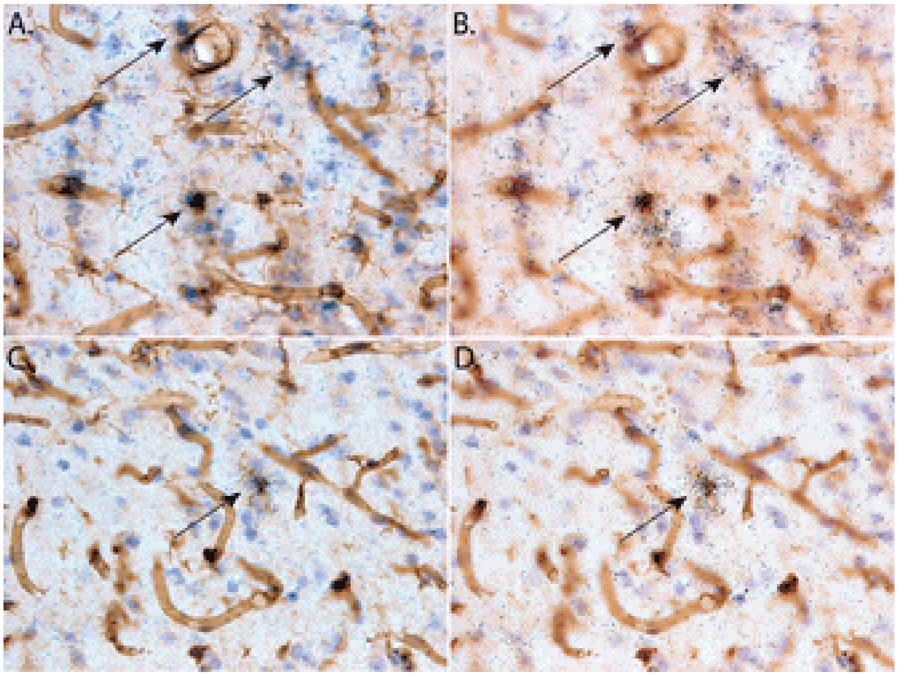

**FIGURE 3.** Golli-MBP expression is differentially expressed in CNS macrophages during the acute and remission phases of EAE. Panels A and B represent histological sections from the cerebellum of a mouse with acute EAE (complete hind limb paralysis). Panels C and D represent histological sections from the cerebellum of a mouse that was in remission from acute EAE. Microglia, macrophages, and blood vessels are visualized in brown with tomato lectin, nuclei are visualized in blue with hematoxylin, and BG21 mRNA expression was visualized by photographic emulsion grains (black) with $^{33}$P label riboprobes. Arrows indicate a Golli-MBP–expressing, lectin+ cells.

In general, the degree of neuroinflammation, microglial activation, and macrophage infiltration is much more robust in EAE than in the rapidly resolving LPS/IFNg model. Thus, it is interesting to note that while stellate, lectin+ cells did express Golli-MBP in the LPS/IFNg model, Golli-MBP+, lectin+ cells had much shorter, thicker process consistent with a highly activated phenotype during the acute stage of EAE. These Golli-MBP+, lectin+ cells were also localized primarily in perivascular sites in this EAE model. In MBP-immunized mice, which had recovered from clinical symptoms, Golli-MBP mRNA expression did not fully return to the untreated or preclinical phenotype even by the time that clinical symptoms had subsided (Fig 3C, D). Rather, Golli-MBP mRNA expression was now associated with occasional stellate-shaped parenchymal macrophages (with morphologies similar to that observed in the LPS/IFNg model) located within the cerebellum and spinal cord.

## What has this Comparative Analysis of Golli-MBP mRNA Expression Revealed about CNS Macrophage Biology?

A few key conclusions can be drawn from these studies. First, cultured microglia and macrophages are important models that can be precisely manipulated, but these cultured models are not always completely predictive of microglial biology within the intact CNS. *In vivo*, the phenotype of CNS-resident microglia and acutely infiltrating macrophages is defined in part by their interactions with CNS neurons. Although the molecular nature of these interactions is only incompletely defined, several neuronally produced molecules, such as CD200, CD22, CCL21 and fractalkine, have demonstrated potential to modify myeloid responses to pathogenic signals[4,24]. *In vitro*, microglia and macrophages lack these phenotype-determining interactions. The difference between *in vitro* and *in vivo* studies also suggests that CNS

insults that alter neuronal function will lead to significant alterations in the phenotype of all CNS macrophage populations[24].

Our data to date are consistent with Golli-MBP expression being enriched in a subset of hematogenously derived macrophages as compared to CNS-resident CD45$^{lo}$ microglia. However, irradiation chimeric studies will be necessary to identify conclusively the identity of the Golli-MBP+ CNS macrophage population. Our data do conclusively illustrate that even within a single pathology, there is significant heterogeneity in CNS macrophage phenotypes and thus likely functions. Future studies will be aimed at determining whether *in vivo* Golli-MBP+ CNS macrophages promote the proliferation and survival of oligodendrocyte and neuronal precursors.

## ACKNOWLEDGMENTS

MJC was supported by grants from NINDS, University of California Riverside (PIC grant program), Biogen Idec, and the Dana Foundation.
